# Analysis of different characteristics of smile

**DOI:** 10.1038/s41405-020-0032-x

**Published:** 2020-05-05

**Authors:** Mehwish Khan, Syed Murtaza Raza Kazmi, Farhan Raza Khan, Imran Samejo

**Affiliations:** 1 Sindh Institute of Oral Health Sciences, Jinnah Sindh Medical University, Karachi, Pakistan; 2 Aga Khan University & Hospital, Karachi, Pakistan

**Keywords:** Dental patient assessment, Fixed prosthodontics, Aesthetic dentistry

## Abstract

**Introduction:**

Analysis of smile is imperative in the diagnosis and treatment planning phases of aesthetic dentistry.

**Aim:**

To evaluate the components of smile among students of a dental institution.

**Methods:**

Frontal view digital photographs with posed smile of 157 dental students were assessed using Adobe Photoshop7.0. Smile characteristics evaluated included; smile line, smile arc, smile design, upper lip curvature, labiodental relationship and number of teeth displayed. Data were analyzed using SPSS version 23.0. Pearson chi-square test was used to determine the gender based differences for various parameters.

**Results:**

Average smile line (43.3%), consonant smile arcs (45.2%), cuspid smiles (45.9%), upward lip curvature (43.9%), maxillary anterior teeth not covered by lower lip (60.5%) and teeth displayed up to first premolars (35.7%). Gender based differences were not statistically significant except for smile arc (*p* value = 0.02) and number of teeth displayed (*p* value < 0.001). There was a significant relationship between lip curvature and smile pattern (*p* value < 0.001) and lip curvature and smile arc (*p* value = 0.01) revealing that upward lip curvature was associated with commissure type smiles and consonant smile arcs.

**Conclusions:**

The smile characteristics should be considered before beginning the aesthetic treatment of the patient to obtain adequate results in oral rehabilitation.

## Introduction

Dento-facial aesthetics has an important role in dental practice of the contemporary era, reflected by increasing demands for more cosmetic and aesthetic procedures by the patients.^1,2^ The perception of beauty varies according to individual preferences and influenced by the ethnic or cultural background.^2^ In order to achieve an optimum aesthetic result in oral rehabilitation, the crucial steps involving proper pretreatment workup, diagnosis and treatment planning cannot be overlooked.^3^

Smile is a facial expression that usually indicates pleasure, friendliness and gratitude.^2^ Analysis of smile is imperative in the diagnosis and treatment planning phases of prosthodontics and aesthetic dentistry. According to neurological control, smile can broadly be divided into involuntary (spontaneous) and voluntary (posed) smile. An involuntary smile is related to emotion, whereas the posed (social) smile is intentional and usually not related with emotion.^4–6^ There are a number of parameters that constitute the natural smile of an individual. These include smile line, smile arc, smile design, upper lip curvature, labiodental relationship, teeth display, buccal corridor, and position of incisal edge. In addition, dental-facial midline, symmetry, gingival display and gingival zenith position also play an important role in the aesthetic appraisal of smile. All these factors must be taken into account while designing a smile makeover. Furthermore, the norms for these smile characteristics may differ in different populations thus the ethnicity should also be taken into account as a variable.

Although, there are abundant data regarding the factors contributing to the smile in the western populations but there is an insufficient data regarding these aesthetic norms among South Asian population. The present study was conducted to evaluate the components of posed smiles in dental students of a private-sector dental institution, which can further be applied as a guide in the fabrication of anterior aesthetic restorations.

## Materials and methods

This cross-sectional study was conducted from December 2017 to May 2018 at a private dental institute in Karachi, Pakistan. The study was approved by the Institutional review board (AUG-2017-PRT05) and written informed consent was obtained from the participants. Sample size was calculated using WHO calculator “Sample size determination in health studies”. A convenience consecutive sampling technique was employed to select total 170 subjects (76 males and 94 females) for the study. Of these 170, four did not give consent to participate in the study and nine were excluded on account of history of orthodontic treatment. Final sample size comprised of 157 (67 males and 90 females) dental students who had healthy natural dentition without any signs of active caries in anterior teeth. In addition to history of orthodontic treatment, subjects with congenital defects, facial asymmetry, maxillofacial trauma, crowding, or severe tooth wear were also excluded.

Standardised posed smile photographs were taken of 157 subjects seated in natural head position using a Nikon camera (D5300 with 105-mm lens (ISO 500, f 1/5.6, exposure time 1/200), fixed in position with a tripod. Each image was assessed using Adobe Photoshop version 7.0.

**Fig. 1:**
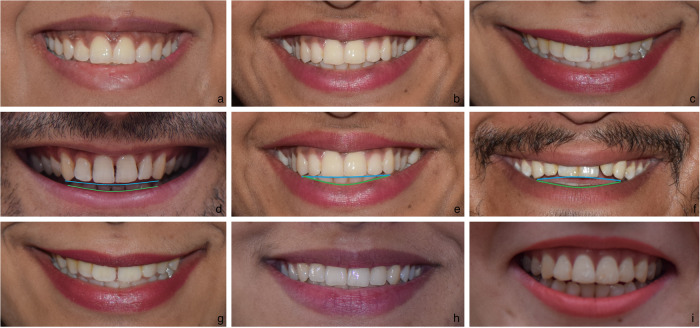
Basic characteristics of smile. (a) High Smile line, (b) Average Smile line, (c) Low Smile line, (d) Consonant Smile arc, (e) Straight Smile arc, (f) Reverse Smile arc, (g) Commissure Smile type, (h) Cuspid Smile type, (i) Complex Smile type.
- Smile arc: is the relationship between the curvature of the maxillary anterior teeth and upper border of the lower lip.^6,8,9^ It was defined in the study by drawing a line along the maxillary central incisal edges to the cusp tips of maxillary canines, which was related to another line drawn across the superior border of the lower lip. In subjects whose maxillary teeth were covered by lower lip, smile arc was designated as "not available". Three categories described: “parallel to the teeth” (when the two lines follow the same curvature) also called as a consonant smile (Fig. 1d). Whereas, if the two are not parallel, it is called a non-consonant smile.^6^ A non-consonant smile can either be “straight” (with flatter curvature of the teeth in relation to the lower lip) (Fig. 1e), or “reverse” (when maxillary teeth form a reverse curve in relation to lower lip) (Fig. 1f).^3^
- Smile design or smile type: three basic patterns have been identified.^10^ The commissure smile (Fig. 1g) is the typical pattern that can be imagined as a Cupid bow. In this pattern, the maxillary first molars lie few millimetres above the incisal edges of the central incisors. In the cuspid smile type (Fig. 1h), the shape of the lips can be demonstrated as a diamond, the rise of the upper lip resembles a window shade. In this design, the position of the maxillary molars is inferior to or at the level of the central incisors. In the complex smile (Fig. 1i), the shape of the lips is visualised as two parallel chevrons, displaying all the upper and lower teeth. The upper lip moves superiorly with the lower lip moving inferiorly during smile.^10^
- Upper lip curvature; a straight line was drawn through the midpoint of the inferior border of the upper lip and its relationship with the corners of mouth evaluated.^1,11^ Three categories identified (Fig. 2e-g); upward (the corners of the mouth lie above the horizontal line), straight (the corners of the mouth at or within 1 mm of the line), and downward (the corners of the mouth lie below the horizontal line).

Following characteristics of smile were evaluated:

- Smile line or lip line: extent of vertical tooth display in smiling or elevation of the upper lip in relation to the maxillary incisors.^7^ Three types have been described, high, average and low^2^ (Fig. 1a–c). High smile that shows the maxillary anterior teeth along with a significant amount of gingiva, average smile showing maxillary anterior teeth with only interproximal gingiva, and low smile that shows less than two-third of the maxillary anterior teeth.

**Fig. 2:**
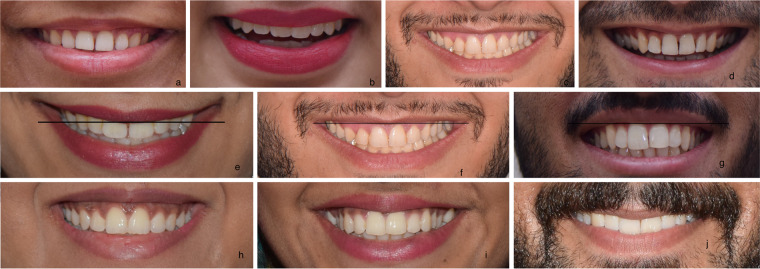
Additional characteristics of smile. (a) Teeth displayed up to canine, (b) Teeth displayed up to first premolars, (c) Teeth displayed up to second premolars, (d) Teeth displayed up to first molars, (e) Upward Upper lip curvature, (f) Straight Upper lip curvature, (g) Downward Upper lip curvature, (h) Lower lip slightly touching the maxillary anterior teeth, (i) Not-touching labiodental relationship, (j) Maxillary anterior teeth covered by lower lip.
- Labiodental relationship of the lower lip and maxillary anterior teeth: was identified by evaluating the distance between the superior border of the lower lip and inferior border of the maxillary anterior teeth. The relationship may be divided into lower lip “slightly touching” the lower border of maxillary anterior teeth (Fig. 2h), “not touching” the teeth(Fig. 2i) or “covering” the anterior teeth (Fig. 2j)^2^
- Number of teeth displayed: Smiles were categorised as displaying teeth up to the canines (Fig. 2a), first premolars (Fig. 2b), second premolars (Fig. 2c), or the first molars (Fig. 2d). A tooth was counted when more than half of its surface was visible.
- In addition, relationship of upper lip curvature with smile arc and different types of smile design was also investigated.

Statistical Package of Social Sciences (SPSS) version 23.0 was used for data analysis. Frequency distribution of the parameters of smile were determined. Pearson Chi-square test was used to determine the gender based differences. Spearman’s correlation coefficient was employed to assess the correlation among parameters of smile in the two genders. Level of significance was kept at 0.05.

## Results

Mean age of males (*n* = 67) and females (*n* = 90) were 22.8 ± 1.6 and 22.5 ± 4 years, respectively (Tables 1–3).

**Table 1:** Smile characteristics of participants (*n* = 157).

Smile parameters	Categories	Frequency (%)
Smile line	Low	61 (38.9%)
	Average	68 (43.3%)
	High	28 (17.8%)
Smile arc	Consonant	71 (45.2%)
	Straight	59 (37.6%)
	Reverse	11 (7.0%)
	Not available	16 (10.2%)
Smile type	Commissure	56 (35.7%)
	Cuspid	72 (45.9%)
	Complex	29 (18.5%)
Upper lip curvature	Upward	69 (43.9%)
	Downward	44 (28.0%)
	Straight	44 (28.0%)
Labiodental relationship	Slightly touching	46 (29.3%)
	Not touching	95 (60.5%)
	Covered by lower lip	16 (10.2%)
Teeth displayed	Canine	32 (20.4%)
	First premolar	56 (35.7%)
	Second premolar	50 (31.8%)
	First molar	19 (12.1%)

**Table 2:** Association of smile parameters with gender.

Smile parameter	*p* value
Smile line	0.791
Smile arc	0.02^*^
Smile type	0.412
Upper lip curvature	0.396
Labiodental relationship	0.589
Teeth displayed	<0.001^*^

*Statistically significant *p* value with chi-square test.

**Table 3:** Assessment of relationship of upper lip curvature with smile arc and smile type.

	Upper lip curvature			*p* value
**Smile arc**	**Upward**	**Downward**	**Straight**	**0.01** ^*^
Consonant	36	18	17	
Straight	15	22	22	
Reverse	06	02	03	
Not available	12	02	02	
**Smile type**	**Upward**	**Downward**	**Straight**	**0.000** ^*^
Commissure	47	06	03	
Cuspid	22	15	35	
Complex	00	23	06	

*Statistically significant *p* value with chi-square test.

Average smile line was frequently observed among the subjects, followed by low smile line. Most common smile type observed was cuspid, followed by commissure and then complex smile type. Upward lip curvature was observed in most individuals followed by equal distribution of downward and straight lip curvatures. No statistically significant gender based difference was observed for smile line, smile type, and upper lip curvature. In addition, most participants had their maxillary anterior teeth not covered by the lower lip during smile without any statistically significant differences between the genders.

For smile arcs, consonant smile arcs were more common followed by flat or straight arc. A statistically significant difference between males and females was observed (*p* value = 0.02) indicating that a consonant smile is common in females and a flat smile in males. Teeth displayed up to first premolars among majority of subjects. With few participants having their first molars visible during smile. Statistically significant gender differences observed in this group (*p* value < 0.001) with males having broader smile displaying teeth up to first molars while females are more likely to display teeth up to first premolars.

Significant association between upper lip curvature and smile design was found (*p* value < 0.001). Commissure smiles were associated with the upward lip curvature whereas, straight lip curvatures were associated with cuspid and downward with gummy smiles. A significant relationship of lip curvature and smile arc (*p* value = 0.01) was also observed, which indicates that more subjects with upward lip curvature were found to have a consonant smile arc.

## Discussion

A detailed examination of the smile characteristics is an essential part of treatment planning in restorative dentistry especially when anterior dentition is involved and the patient has high aesthetic demands. The present study has explored common features of a posed smile among a sample of Pakistani students. Posed smile was employed in the present study mainly because it’s readily reproducible. The common findings were: average smile line, consonant smile arcs, cuspid-type smile, an upward lip curvature, with a not-touching relationship of maxillary anterior teeth with lower lip and teeth displayed up to first premolars.

Consideration of smile line has a clinical application in treatment of the patients, great care should be taken to avoid excessive display of gingiva during restoration of anterior teeth in patients with high smile lines. Average smile line was observed to be the most frequent among participants in the present study. Other studies such as Tjan et al.^2^ and Nold et al.^3^ have also reported similar findings. Tjan and Miller^2^ also reported that high smile line were least common in their study, this too is in agreement with the present study. Contrary to this, Nold et al.^3^ revealed low smile line to be least common among their study participants. Furthermore, aforementioned studies showed statistically significant differences between male and female participants for the position of smile line. However, no such difference has been observed in the present study. Clinically, it is known that low smile lines are more tolerant to inadequacies in the anterior restorations and hence it is easier for the dentist to satisfy these patients with the restorative dentistry work.^12^

The term smile arc has different definitions in literature of Prosthodontics, Orthodontics and Cosmetic dentistry.^9^ In the present study, smile arc has been defined as described by Sarver.^9^ A consonant smile arc is considered to be more attractive than a non-consonant smile.^6^ Consonant smile arc was most commonly observed among the participants of this study whereas reverse smile arc was least frequent. These findings are in agreement with Tjan and Miller,^2^ Nold et al.,^3^ and Desai et al.^13^ Statistically significant difference existed regarding smile arc between the two genders, this was also testified by Nold et al.^3^ On the other hand, Maulik and Nanda^14^ reported that straight smile arc was the most common finding observed in 49% of their participants followed by consonant and reverse smile arc in 40% and 10% subjects, respectively. Their methodology involved capturing spontaneous smiles by making video of orthodontically treated and untreated subjects. This may be the reason of different results from our study which involved posed smile of otherwise healthy subjects.

A large proportion of the subjects had cuspid smile in the present study, whereas previous studies^10,15,16^ have reported the commissure smile pattern as the most common. Liang et al. in their study on Chinese subjects reported that more female subjects were found to have commissure smile pattern while complex smile being primarily a male feature.^15^ The recent study did not show any gender based differences regarding the smile pattern. These differences may be attributed to the difference in population studied.

According to Hulsey;^8^ an upward lip curvature was the most prevalent feature in subjects of his study, which is similar to the present study. Contrary to this, Liang et al.^15^ showed large number of individuals with straight lip curvatures, followed by downward and upward. These contradictory results stem out to demographic variation. Dong et al.^11^ reported that straight to upward lip curvatures are considered more appealing than downwards. It has been documented that curvature of the upper lip cannot be altered by orthodontic treatment, thus attaining an ideal smile in a patient with downward lip curvature is limited.^7^

Majority of the participants in the present study, had a non-touching labiodental relationship followed by relatively few participants with covering and slightly touching relationships (with no gender based difference statistically); these results are similar to that reported by Nold et al.^3^ This is contradictory to the findings of Tjan et al.^2^. They have observed a higher percentage of touching labiodental relationship in their studies. Moreover, significant differences in males and females were also recorded. Desai et al.^13^ reported that with increasing age, people usually cover their maxillary incisal edges with their lower lip during smile. Dong et al.^11^ concluded that greater aesthetic scores were obtained for patients whose lower lips slightly touched or did not touch the maxillary anterior teeth than those whose teeth were covered by lower lip. Moreover, everted lower lip is usually seen with excessive proclination of incisors, while upright and retroclined incisors are partially covered by the lower lip.^7^

Data of the present study suggest that individuals usually display six maxillary anterior teeth along with the first premolars during posed smile. Dong et al and Maulik and Nanda observed teeth display upto second premolars during smile.^11,14^ Similar to our study, teeth display upto first premolar was also reported by Tjan et al.^2^ However, Nold et al.^3^ showed that only 24% participants (all Caucasians) showed teeth up to the first premolar on posed smile. Both these studies showed no gender based differences for extent of teeth visible during smile. And, the present study suggests that males have wider smiles, exposing more teeth than females. Greater smile width among males requires consideration during treatment planning of anterior restorations.

A correlation between pattern of smile design and upper lip curvature was observed in the present study. Most participants with upward lip curvature had a commissure smile type whereas subjects with cuspid smiles showed predominantly straight lip curvatures. Downward upper lip curvature was primarily seen in individuals with gummy smiles. A similar association was reported by Liang et al.^15^ where straight or upward upper lip curvatures were seen predominantly with commissural smiles, and downward upper lip curvature among subjects with cuspid type and gummy smiles.

This study also proposes a relationship between consonant smile arcs and upward lip curvature, which suggests that curvature of the upper lip may affect the position of smile arc. More research is needed to confirm if such an association among different characteristics of smile can be established. One limitation of the present study lies in its lack of generalizability. As this study was conducted in only one dental school located in the largest city of the country but it limits the generalizability of the results to the whole population. In addition, there was no information on occlusion, cephalometric/ anthropometric measurements of maxilla and mandible. A large sample size comprising of ethnically diverse group of subjects are warranted with inclusion of some other components contributing to smile such as occlusal plane, symmetry, lip length, lip thickness and smile width and gingival zenith etc.

## Conclusions

Within the limitations of the study, it can be concluded that the findings in the studied sample were presence of average smile line, consonant smile arcs, cuspid-type smile, an upward lip curvature, with a non-touching relationship of maxillary anterior teeth with lower lip and teeth displayed up to first premolars. These findings can be incorporated in smile analysis of individuals belonging to the South Asian population before beginning the aesthetic treatment. Moreover, each case must be assessed individually along with the consideration of the expectations and preferences of the patient to obtain adequate results in oral rehabilitation.

## References

[1] Al-Johany SS, Alqahtani AS, Alqahtani FY, Alzahrani AH (2011). Evaluation of different esthetic smile criteria. Int J. Prosthodont..

[2] Tjan AH, Miller GD, The JG (1984). Some esthetic factors in a smile. J. Prosthet. Dent..

[3] Nold SL, Horvath SD, Stampf S, Blatz MB (2014). Analysis of select facial and dental esthetic parameters. Int J. Periodont. Restor. Dent..

[4] Gill DS, Naini FB, Tredwin CJ (2007). Smile aesthetics. Dent. Updat..

[5] Rigsbee OH, Sperry TP, BeGole EA (1988). The influence of facial animation on smile characteristics. Int. J. Adult Orthodon. Orthognath. Surg..

[6] Sarver DM, Ackerman MB (2003). Dynamic smile visualization and quantification: Part 2. Smile analysis and treatment strategies. Am. J. Orthod. Dentofac. Orthop..

[7] Sabri R (2005). The eight components of a balanced smile. J. Clin. Orthod..

[8] Hulsey CM (1970). An esthetic evaluation of lip-teeth relationships present in the smile. Am. J. Orthod..

[9] Sarver DM (2001). The importance of incisor positioning in the esthetic smile: the smile arc. Am. J. Orthod. Dentofac. Orthop..

[10] Rubin LR (1974). The anatomy of a smile: its importance in the treatment of facial paralysis. Plast. Reconstr. Surg..

[11] Dong JK, Jin TH, Cho HW, Oh SC (1999). The esthetics of the smile: a review of some recent studies. Int J. Prosthodont..

[12] Mehta SB, Banerji S, Aulakh R (2015). Patient assessment: preparing for a predictable aesthetic outcome. Dent. Updat..

[13] Desai S, Upadhyay M, Nanda R (2009). Dynamic smile analysis: changes with age. Am. J. Orthod. Dentofac. Orthop..

[14] Maulik C, Nanda R (2007). Dynamic smile analysis in young adults. Am. J. Orthod. Dentofac. Orthop..

[15] Liang L-Z, Hu W-J, Zhang Y-L, Chung K-H (2013). Analysis of dynamic smile and upper lip curvature in young Chinese. Int J. Oral. Sci..

[16] Patterns IS (1999). The classification of smile patterns. J. Can. Dent. Assoc..

